# Switchable Coupled Relays Aid Massive Non-Orthogonal Multiple Access Networks with Transmit Antenna Selection and Energy Harvesting

**DOI:** 10.3390/s21041101

**Published:** 2021-02-05

**Authors:** Thanh-Nam Tran, Miroslav Voznak

**Affiliations:** 1 Faculty of Electrical Engineering and Computer Science, VSB-Technical University of Ostrava, 17. Listopadu 2172/15, 708 00 Ostrava, Czech Republic; miroslav.voznak@vsb.cz; 2 Faculty of Electronics and Communications, Sai Gon University, 273 An Duong Vuong, Hochiminh 700 00, Vietnam

**Keywords:** non-orthogonal multiple access, multiple devices, multiple antennas, massive MIMO, transmit antenna selection, energy harvesting

## Abstract

The article proposes a new switchable coupled relay model for massive MIMO-NOMA networks. The model equips a much greater number of antennas on the coupled relays to dramatically improve capacity and Energy Efficiency (EE). Each relay in a coupled relay is selected and delivered into a single transmission block to serve multiple devices. This paper also plots a new diagram of two transmission blocks which illustrates energy harvesting and signal processing. To optimize the system performance of a massive MIMO-NOMA network, i.e., Outage Probability (OP) and system throughput, this paper deploys a Transmit Antenna Selection (TAS) protocol to select the best received signals from the pre-coding channel matrices. In addition, to achieve better EE, Simultaneously Wireless Information Power Transmit (SWIPT) is implemented. Specifically, this paper derives the novel theoretical analysis in closed-form expressions, i.e., OP, system throughput and EE from a massive MIMO-NOMA network aided by switchable coupled relays. The theoretical results obtained from the closed-form expressions show that a massive MIMO-NOMA network achieves better OP and greater capacity and expends less energy than the MIMO technique. Finally, independent Monte Carlo simulations verified the theoretical results.

## 1. Introduction

Relays are proven suitable solutions for extending networking coverage. In addition, Multiple-Input–Multiple-Output (MIMO) Non-Orthogonal Multiple Access (NOMA) technologies are effective methods for enhancing the capacity of fifth-generation (5G) wireless communication networks and beyond. The NOMA technique improves spectral sharing and the capability for a greater number of connections in the same time slot/frequency [1,2]. The NOMA technique in fifth-generation (5G) wireless communication networks and beyond could therefore serve large numbers of devices. The NOMA technique’s feature is the ability to broadcast a superimposed signal to devices in the network with different Power Allocation (PA) factors [3]. The farthest device with poorest Channel State Information (CSI) is allocated the largest PA factor compared to other PA factors allocated to other devices. Successive Interference Cancellation (SIC) [4] implemented at the receiver device removes interference before the device decodes its own message in the superimposed signal by treating other device information which has a stronger PA factor [5]. Major studies have significantly contributed to NOMA through the application of new techniques in 5G networks and beyond. In [6], the authors verified that the system performance of NOMA networks is dependent on power resource allocation. The main aims of NOMA networks are to serve more devices [7] and fairness in Quality of Service (QoS) [8]. In achieving these aims, previous studies verify that the system performance of NOMA wireless networks are significantly affected by power resource allocation strategies [9,10]. The authors verified that PA factors in NOMA networks could be allocated by device CSI and device data rate thresholds [11].

Recently, a cooperative networking solution has drawn much attention as an emerging method to extend network coverage. Specifically, in a cooperative NOMA network, a relay is deployed to receive and forward superimposed signals to the devices outside the network area. The radius of the network coverage is thus expanded, and its reliability is improved by enhancing QoS for devices [12]. Some major techniques may be fully deployed at the relay, namely Half-Duplex (HD), Full-Duplex (FD), Decode-and-Forward (DF), and Amplify-and-Forward (AF) [13]. Some studies have made significant contributions to cooperative NOMA [14,15]. These studies have shown that the system performance of NOMA wireless networks can be improved by deploying multiple relays in combination with the selection of the best relay in individual schemes [16,17,18,19].

In addition, to improve the capacity of a NOMA wireless network, the massive MIMO technique [20] is emerging as a good solution which extends the MIMO technique [21,22]. However, under this technique, networked devices equip more antennas, leading to increased costs for hardware and RF. The authors proposed the deployment of a TAS protocol which selects the best channel [23,24]. The authors also designed the pre-coding matrices for optimal system throughput in a multi-cell MIMO-NOMA network. However, massive NOMA has many research challenges which require careful investigation, such as extending the massive MIMO network which consider relaying and TAS and SWIPT protocols.

**Figure 1:**
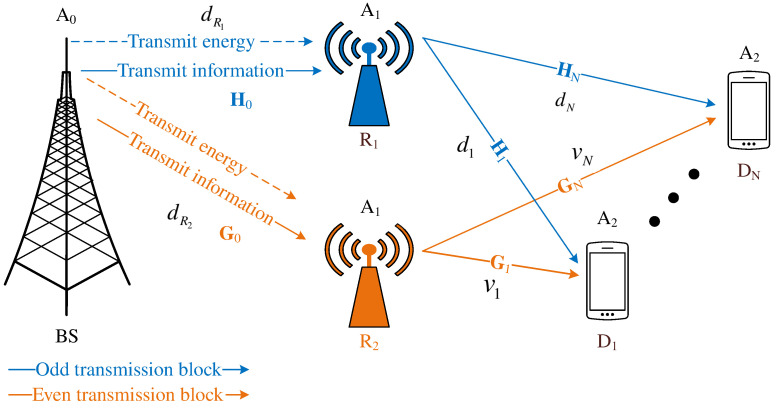
Coupled relays in a cooperative MIMO-NOMA network with the application of TAS and SWIPT.

**Figure 2:**
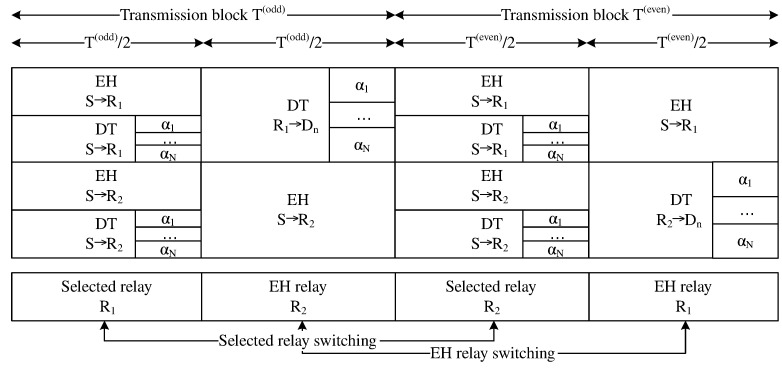
Diagram of two transmission blocks.

**Figure 3:**
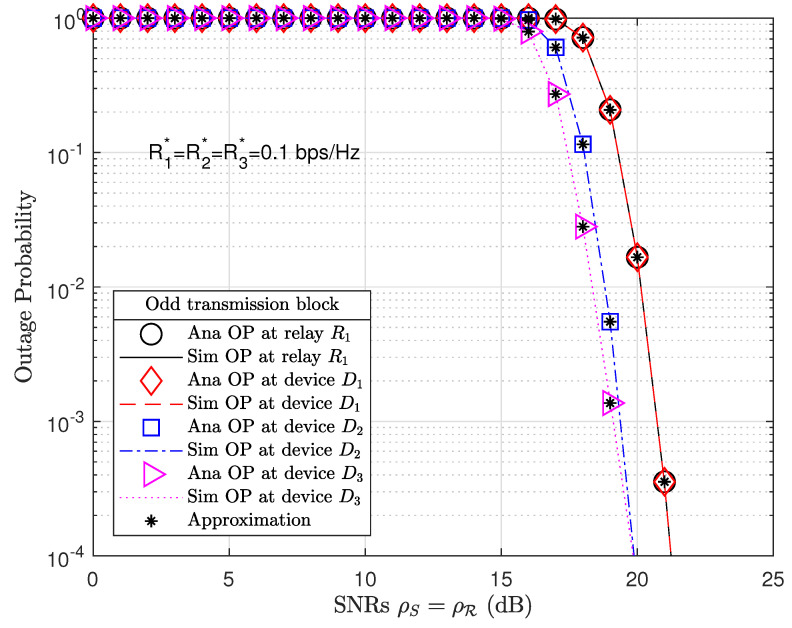
OP at relay $R_{1}$ and devices $D_{n}$ for $n=\left\{1,\ldots ,N\right\}$ in an odd transmission block, where the number of antennas equipped at the BS, relay $R_{1}$ and devices $D_{n}$ are $A_{0}=4$, $A_{1}=4$, and $A_{2}=2$, respectively, and the PS factor λ=0.4.

Another potential technology for future 5G networks and beyond is the simultaneous transmission of information with Radio Frequency (RF) and Energy Harvesting (EH) [25,26,27,28,29,30,31]. A study [26] on wireless EH offers a deep survey of the advantages of SWIPT. The authors surveyed several SWIPT technologies: SWIPT enabled multi-carrier systems, full-duplex SWIPT systems, etc. Given the explosion in the number of networked devices, for example Internet of Things (IoTs) devices, the energy issue is especially important. Time Switching (TS) [28] and Power Splitting (PS) [29,30,31] represent a solution for simultaneous data and energy transmission. Previous studies [28,29,30,31] which contained some problems requiring further investigation were the motivations which led to this study. In [28], the authors proposed a MIMO system consisting of a single transmitter which served a single receiver. The authors adopted the TS framework as in [28] (Figure 2), and the framework was split into two time slots. The first time slot was used for the transmission of wireless power [28] (Equation (3)), and the second time slot was used for the transmission of wireless information [28] (Equation (4)). However, the authors adopted PS frameworks for cooperative NOMA networks to assist the far device through a single relay equipped with a single antenna [29,30,31]. The authors deployed multiple relays to aid a single destination [31] (Figures 1a and 3a) by adopting TS and PS frameworks [31] (Figures 1b and 3b), respectively.

This study was inspired by the major studies [29,31]. The authors of [31] adopted a SWIPT protocol and depicted TS and PS frameworks fully. However, the system models in [31] were only designed to serve a single destination whereas the authors of [29] deployed a cooperative NOMA network to serve multiple devices (two devices). However, the author of [29] equipped a single antenna on each network node. The authors of the present study designed a network to serve a greater number of devices than the number of devices in [29,31] and improved networking capacity by equipping a greater number of antennas on network nodes, i.e., BS, coupled relays, and devices.

The study’s main aims are also its major contributions:

- We present a new design for a switchable coupled relay model to assist massive MIMO-NOMA wireless networks. Each relay in a coupled relay is selected and delivered into odd/even transmission blocks. The selected relay is used to forward signals to multiple devices while another relay maintains EH.
- We present a new design for a diagram of two transmission blocks to calculate the propagation of wireless information and power (WIP). The present paper offers the potential for the practical application of wireless sensor networks (WSNs), e.g., in a water environment where relays and devices are barely powered [32].
- We maximize system throughput in a massive MIMO-NOMA network. The study deploys a TAS protocol which selects the best received signals from the pre-coding channel matrices.
- The study delivers novel expressions for OP, system throughput, and EE in closed-forms. We apply Monte Carlo simulation results to verify the analysis results.

This paper is structured as follows. Section 2 introduces system modeling. Section 3 presents an analysis of the system model. We describe and discuss the analysis and simulation results in Section 4. Section 5 presents a summary of the paper.

## 2. System Model

### 2.1. New Design for a Cooperative MIMO-NOMA Scheme

The present paper examines a new cooperative MIMO-NOMA model for emerging 5G wireless networks and networks beyond. Figure 1 depicts the system model containing a BS, coupled relay $R=\left\{R_{1},R_{2}\right\}$, and multiple devices $D_{n}=\left\{D_{1},\ldots ,D_{N}\right\}$. To benefit a massive MIMO-NOMA network, the study equips a large number of antennas $A_{0}$, $A_{1}$, and $A_{2}$ at the network nodes and BS, coupled relays, and devices, respectively. In addition, we assume that the BS has full knowledge of the CSI [26,33]. As with the system models in the major studies [28,29,30,31], the massive MIMO underlay cooperative NOMA model shown in Figure 1 contains two time slots to complete a transmission block. It is important to illustrate the difference to previous major studies. In [16,17,18,19], the authors verified that relays are a good solution to assist in combating channel fading. In other major studies, the authors deployed a SISO relay to aid a multi-destinations [29] or a multiple SISO relay to aid a single destination [31]. The authors also proposed relay selection strategies to select the best nearest relay [17] and max-instantaneous data rate [18] (Equation (16)) to assist the destinations. The present study deploys coupled relays to assist massive MIMO-NOMA networks, however, only the relay with better power capacity among the coupled relays is selected for cooperating devices in an odd/even transmission block., i.e., while one relay is selected to forward the signal to devices, another relay has to maintain EH from the BS.

Note that the present study is designed to serve multiple devices simultaneously. The coupled relays must first have the device CSI and report this information to the BS. Based on the device CSI, the BS allocates PS factors, whereas the coupled relays feed back their own CSIs to the BS and other devices. Based on the energy capacity information, the BS selects the best powered relay to forward the signal. The devices then wait to receive the signal from the strongest powered relay. In the case of insufficient CSI, the BS may select the poorest powered relay to forward the signal, leading to a reduced lifespan of the relay. This may interrupt signal propagation because the devices are waiting for a non-cooperative relay.

### 2.2. Propagation and Formulations

From the model depicted in Figure 1, we designed a new propagation diagram, as shown in Figure 2. The coupled relays feed back data to the BS about their energy capacities. The BS decides which relay has more energy to forward the superimposed signal, while the remaining relay has less energy to maintain EH. Two main phases take place: EH and data transmission (DT). To illustrate, Figure 2 depicts two transmission blocks: an odd transmission block $T^{\left(odd\right)}$ and an even transmission block $T^{\left(even\right)}$. Each transmission block $T_{\;}^{\left(\theta \right)}$ for $\theta =\left\{odd,even\right\}$ is separated into two equal time slots. The first time slot T1odd=ToddTodd22 in odd transmission block $T^{\left(odd\right)}$ is used by the BS to transmit wireless energy and superimposed information to coupled relays $R=\left\{R_{1},R_{2}\right\}$. In terms of the SWIPT technique with a PS protocol, during the first time slot $T_{1}^{\left(odd\right)}$, a fraction λ of the power domain $P_{S}$ is used for EH while the remaining fraction (1−λ) of the power domain $P_{S}$ is used to superimpose data from the BS. The second time slot T2odd=ToddTodd22 in odd transmission block $T^{\left(odd\right)}$ is used by the best powered relay to forward the superimposed signal to devices, while the worse powered relay applies EH from the BS, where the PS factor λ=1. The present study assumes that relay $R_{1}$ is selected in the odd transmission block because relay $R_{1}$ has more energy than relay $R_{2}$. Therefore, relay $R_{2}$ maintains EH from the BS. Similarly, the even transmission block $T^{\left(even\right)}$ is also separated into two equal time slots. The first time slot T1even=TevenTeven22 in even transmission block $T^{\left(even\right)}$ is also used by the BS to simultaneously transmit wireless energy and the superimposed signal to the coupled relay $R=\left\{R_{1},R_{2}\right\}$. However, in the second time slot T2even=TevenTeven22 in even transmission block $T^{\left(even\right)}$, the relay $R_{2}$ is selected to forward the superimposed signal to devices instead of relay $R_{1}$ because relay $R_{2}$ harvested energy from the BS, where λ=1 during $T_{2}^{\left(odd\right)}$, and then relay $R_{2}$ contains more energy than relay $R_{1}$. As a result, relay $R_{1}$ maintains EH from the BS with PS factor λ=1 during $T_{2}^{\left(even\right)}$. Although the network model shown in Figure 1 is complex, it requires two time slots for signals to propagate through the network as in studies [28,29,31]. Specifically, the present study examines how multiple devices are served simultaneously. We therefore adopt the emerging NOMA technique. The BS also superimposes all the information of devices in the same signal by sharing the spectrum. The devices may be thus served simultaneously. As a result, the massive MIMO-NOMA network in the present study are low latency.

#### 2.2.1. Odd Transmission Block

As shown in Figure 1, it is important to note that both the BS and coupled relays ($R_{1}$ and $R_{2}$) are equipped with multiple antennas, where $A_{0}>1$ and $A_{1}>1$. $A_{0}$ and $A_{1}$ are the number of antennas at the BS and coupled relays, respectively. Tran et al. [23] designed a pre-coding channel matrix size of [number of transmitting antennas × number of receiving antennas]. We therefore designed the pre-coding channel matrices from the $A_{0}$ transmitting antennas at the BS to the $A_{1}$ receiving antennas on the coupled relays $R_{1}$ and $R_{2}$, respectively, as follows:

(1) $$\begin{array}{c} H_{0}=\left[\begin{array}{ccc} h_{0}^{\left(1,1\right)} & \cdots & h_{0}^{\left(1,A_{1}\right)}\\ \vdots & \ddots & \vdots \\ h_{0}^{\left(A_{0},1\right)} & \cdots & h_{0}^{\left(A_{0},A_{1}\right)} \end{array}\right], \end{array}$$

(2) $$\begin{array}{c} G_{0}=\left[\begin{array}{ccc} g_{0}^{\left(1,1\right)} & \cdots & g_{0}^{\left(1,A_{1}\right)}\\ \vdots & \ddots & \vdots \\ g_{0}^{\left(A_{0},1\right)} & \cdots & g_{0}^{\left(A_{0},A_{1}\right)} \end{array}\right], \end{array}$$

where $h_{0}^{\left(a_{0},a_{1}\right)}\in H_{0}$ and $g_{0}^{\left(a_{0},a_{1}\right)}\in G_{0}$ for $a_{0}\in A_{0}$ and $a_{1}\in A_{1}$ are channels from a transmitting antenna $a_{0}$ at the BS to a receiving antenna $a_{1}$ at the coupled relays $R_{1}$ and $R_{2}$. In addition, the fading channels are modeled over Rayleigh distributions with $h_{0}^{\left(.,.\right)}$ and $g_{0}^{\left(.,.\right)}$ following $h_{0}^{\left(.,.\right)}=d_{R_{1}}^{-\omega }$ and $g_{0}^{\left(.,.\right)}=d_{R_{2}}^{-\omega }$, where $d_{R_{1}}$ and $d_{R_{2}}$ are the distances from the BS to coupled relays $R_{1}$ and $R_{2}$, respectively, and the coefficient ω is the path-loss exponent factor.

By applying the PS protocol, the first time slot in the odd transmission block $T_{1}^{\left(odd\right)}$ is used for the BS to transmit wireless energy and superimposed information simultaneously. To illustrate, two phases take place. In the first phase, the BS sends wireless energy to the coupled relay with the PS factor λ. Therefore, the EH from the best channel in the pre-coding channel matrices given by (1) and (2) in the first time slot $T_{1}^{\left(odd\right)}$ in odd transmission block $T^{\left(odd\right)}$ at coupled relays $R_{1}$ and $R_{2}$ are expressed as follows:

(3) $$\begin{array}{c} E_{R_{1}}^{\left(T_{1}^{\left(odd\right)}\right)}=\eta \lambda P_{S}\max_{\left[A_{0}\times A_{1}\right]}\left\{{\left|H_{0}\right|}^{2}\right\}, \end{array}$$

(4) $$\begin{array}{c} E_{R_{2}}^{\left(T_{1}^{\left(odd\right)}\right)}=\eta \lambda P_{S}\max_{\left[A_{0}\times A_{1}\right]}\left\{{\left|G_{0}\right|}^{2}\right\}, \end{array}$$

where η is the collection factor, λ is the PS factor, and $P_{S}$ is the transmission power at the BS.

In the second phase of the first time slot $T_{1}^{\left(odd\right)}$ in odd transmission block $T^{\left(odd\right)}$, in a major advantage of NOMA theories, the BS broadcasts a superimposed signal by superimposing the messages $x_{i}$ of devices $D_{i}$ for $i=\left\{1,\ldots ,N\right\}$ to the coupled relays $R_{1}$ and $R_{2}$. We assume that no direct down-link exists from the BS to the devices. The received signal at the coupled relays is expressed as follows:

(5) $$\begin{array}{c} \max_{\left[A_{0}\times A_{1}\right]}\left\{Y_{R_{1}}^{\left(T_{1}^{\left(odd\right)}\right)}\right\}=\sqrt{1-\lambda }\max_{\left[A_{0}\times A_{1}\right]}\left\{\left|H_{0}\right|\right\}\sum_{i=N}^{1}\sqrt{\alpha_{i}P_{S}}x_{i}+n_{R_{1}}, \end{array}$$

(6) $$\begin{array}{c} \max_{\left[A_{0}\times A_{1}\right]}\left\{Y_{R_{2}}^{\left(T_{1}^{\left(odd\right)}\right)}\right\}=\sqrt{1-\lambda }\max_{\left[A_{0}\times A_{1}\right]}\left\{\left|G_{0}\right|\right\}\sum_{i=N}^{1}\sqrt{\alpha_{i}P_{S}}x_{i}+n_{R_{2}}, \end{array}$$

where $n_{R}∼CN\left(0,N_{0}\right)$ is the Additive White Gaussian Noise (AWGN) at the coupled relays $R=\left\{R_{1},R_{2}\right\}$ with zero mean and variance $N_{0}$. The PA factors for devices $D_{i}$ for $i=\left\{N,\ldots ,1\right\}$ are, respectively, denoted by $\alpha_{i}$, constrained to $\alpha_{1}<\ldots <\alpha_{N}$, $\alpha_{1}+\ldots +\alpha_{N}=1$, and given by

(7) αi=ii∑n=1Nn∑n=1Nn.

Note that Expression (7) is derived from the feature studied [7]. However, the devices in [7] were ordered where $D_{1}$ is the farthest device. However, the system model shown Figure 1 indicates that device $D_{N}$ has the farthest distance from the coupled relays. In term of NOMA theory, the farthest device must be allocated the biggest PA factor. As a result, the PA factor $\alpha_{N}$ for device $D_{N}$ is the largest value among the PA factors, whereas device $D_{1}$ is the nearest distance from the coupled relays. Therefore, device $D_{1}$ is allocated the smallest PA factor.

SIC is another feature of NOMA theories which is implemented at the user. The user therefore implements SIC to detect messages in the received signal. In [7,12], the authors investigated NOMA networks with a random number *N* of users. The users repeated the SIC phases until their own messages were successfully detected in the received signal. It is important to note that *N* devices exist in our model (Figure 1), and, therefore, *N* SIC phases at the relay $R_{1}$. After selecting the received signal as given in (5), in the first SIC phase, relay $R_{1}$ detects the message $x_{N}$ of device $D_{N}$ as a result of the constraint of the PA factors $\alpha_{N}>\ldots >\alpha_{1}$. In the second SIC phase, relay $R_{1}$ detects the message $x_{N-1}$ of device $D_{N-1}$ after removing the $x_{N}$ symbol from the received signals. The relay $R_{1}$ repeats SIC until it successfully detects the last symbol $x_{1}$.

However, the present study examines a massive MIMO underlay cooperative NOMA network in contrast to the schemes presented in [29,31], where the author studied cooperative Single-Input-Single-Output (SISO)-NOMA schemes. Fortunately, the authors of [23,24] also investigated a MIMO-NOMA network with TAS and obtained Signal-to-Interference-plus-Noise Ratios (SINRs), where the devices detected information by applying SIC. To optimize system performance, our study considers the massive MIMO technique in combination with a TAS protocol, where relay $R_{1}$ selects the best received signal $\max\left\{\left|Y_{R_{1}}^{\left(T_{1}^{\left(odd\right)}\right)}\right|\right\}$ for SIC. In the first SIC phase, relay $R_{1}$ decodes the $x_{N}$ symbol from the best received signal $\max\left\{\left|Y_{R_{1}}^{\left(T_{1}^{\left(odd\right)}\right)}\right|\right\}$ by treating the data symbols $x_{j}=\left\{x_{1},\ldots ,x_{N-1}\right\}$ and AWGN $n_{R_{1}}$ as interference. The SINR is therefore obtained when relay $R_{1}$ decodes the $x_{i}$ symbol, as follows:

(8) $$\begin{array}{cc} \max_{\left[A_{0}\times A_{1}\right]}\left\{\underset{i=\left\{N,\ldots ,1\right\}}{\gamma_{R_{1}-x_{i}}^{\left(T_{1}^{\left(odd\right)}\right)}}\right\} & =\frac{\left(1-\lambda \right)\max_{\left[A_{0}\times A_{1}\right]}\left\{{\left|H_{0}\right|}^{2}\right\}\alpha_{i}\rho_{S}}{\left(1-\lambda \right)\max_{\left[A_{0}\times A_{1}\right]}\left\{{\left|H_{0}\right|}^{2}\right\}\rho_{S}\sum_{j=1}^{i-1}\alpha_{j}+1},\mathrm{for}\;i>1, \end{array}$$

(9) $$\begin{array}{cc} \; & =\left(1-\lambda \right)\max_{\left[A_{0}\times A_{1}\right]}\left\{{\left|H_{0}\right|}^{2}\right\}\alpha_{i}\rho_{S},\;\;\;\mathrm{for}\;i=n=1, \end{array}$$

where $\rho_{S}$ is the transmission Signal-to-Noise Ratio (SNR) and ρ0=PSPSN0N0.

Maximization of the instantaneous bit rate threshold is achieved at relay $R_{1}$ when relay $R_{1}$ decodes the message $x_{i}$ for $i=\left\{N,\ldots ,1\right\}$ as follows:

(10) $$\begin{array}{c} \max_{\left[A_{0}\times A_{1}\right]}\left\{\underset{i=\left\{N,\ldots ,1\right\}}{R_{R_{1}-x_{i}}^{\left(T_{1}^{\left(odd\right)}\right)}}\right\}=\frac{1}{2}\log_{2}\left(1+\max_{\left[A_{0}\times A_{1}\right]}\left\{\underset{i=\left\{N,\ldots ,1\right\}}{\gamma_{R_{1}-x_{i}}^{\left(T_{1}^{\left(odd\right)}\right)}}\right\}\right). \end{array}$$

In the second time slot $T_{2}^{\left(odd\right)}$ in the odd transmission block $T^{\left(odd\right)}$, the relay $R_{1}$ retrieves the messages $x_{i}=\left\{x_{N},\ldots ,x_{1}\right\}$ and forwards the messages to the devices in the superimposed signal while the relay $R_{2}$ continues harvesting energy from the BS. Therefore, the received signal at devices $D_{n}$ for $n=\left\{1,\ldots ,N\right\}$ and the EH at relay $R_{2}$ are expressed, respectively, as follows:

(11) $$\begin{array}{c} \max_{\left[A_{1}\times A_{2}\right]}\left\{Y_{n\in N}^{\left(T_{2}^{\left(odd\right)}\right)}\right\}=\max_{\left[A_{1}\times A_{2}\right]}\left\{\left|H_{n}\right|\right\}\sum_{i=N}^{1}\sqrt{\alpha_{i}P_{R_{1}}}x_{i}+n_{n}, \end{array}$$

(12) $$\begin{array}{c} E_{R_{2}}^{\left(T_{2}^{\left(odd\right)}\right)}=\eta \lambda P_{S}\max_{\left[A_{0}\times A_{1}\right]}\left\{{\left|G_{0}\right|}^{2}\right\}, \end{array}$$

where $P_{R_{1}}$ is the transmission power at relay $R_{1}$ and $n_{n}∼CN\left(0,N_{0}\right)$ is the AWGN at device $D_{n}$ for $n=\left\{1,\ldots ,N\right\}$ which follows zero mean and variance $N_{0}$.

Note that the pre-coding channel matrix $H_{n}$ is given by

(13) $$\begin{array}{c} \underset{n=\left\{1,\ldots ,N\right\}}{H_{n}}=\left[\begin{array}{ccc} h_{n}^{\left(1,1\right)} & \cdots & h_{n}^{\left(1,A_{2}\right)}\\ \vdots & \ddots & \vdots \\ h_{n}^{\left(A_{1},1\right)} & \cdots & h_{n}^{\left(A_{1},A_{2}\right)} \end{array}\right], \end{array}$$

where the channel $h_{n}^{\left(a_{1},a_{2}\right)}$ in the pre-coding channel matrix $H_{n}$, where $a_{1}\in A_{1}$ and $a_{2}\in A_{2}$, is a channel from transmitting antenna $a_{1}$ at relay $R_{1}$ to a receiving antenna $a_{2}$ at a device $D_{n}$, also applying Rayleigh distribution for propagation. Each fading channel gain is given by $h_{n}^{\left(.,.\right)}=d_{n}^{-\omega }$, where $d_{n}$ is the distance from relay $R_{1}$ to device $D_{n}$.

As a result of the combination of TAS and SIC, the SINRs are obtained at devices $D_{n}$ for $n=\left\{1,\ldots ,N\right\}$ when the devices decode data symbols $x_{i}$ for $i=\left\{N,\ldots ,n\right\}$ from the best received signal $\max{\left\{\left|Y_{2}\right|\right\}}_{A_{1}\times A_{2}}$ by treating the data symbols $x_{j}$ for $j=\left\{1,\ldots ,i-1\right\}$ and AWGN $n_{n}$ as interference:

(14) $$\begin{array}{cc} \max_{\left[A_{1}\times A_{2}\right]}\left\{\gamma_{\underset{n\in N}{\;n}-\underset{i=\left\{N,\ldots ,n\right\}}{x_{i}}}^{\left(T_{2}^{\left(odd\right)}\right)}\right\} & =\frac{\max_{\left[A_{1}\times A_{2}\right]}\left\{{\left|H_{n}\right|}^{2}\right\}\alpha_{i}\rho_{R_{1}}}{\max_{\left[A_{1}\times A_{2}\right]}\left\{{\left|H_{n}\right|}^{2}\right\}\sum_{j=1}^{i-1}\alpha_{j}\rho_{R_{1}}+1},\mathrm{for}\;i>1, \end{array}$$

(15) $$\begin{array}{cc} \; & =\max_{\left[A_{1}\times A_{2}\right]}\left\{{\left|H_{n}\right|}^{2}\right\}\alpha_{i}\rho_{R_{1}},\;\;\mathrm{for}\;i=n=1, \end{array}$$

where SNR ρR1=PR1P1N0N0.

The achievable bit-rate reached at device $D_{n}$ when it decodes the data symbol $x_{i}$ for $i=\left\{N,\ldots ,n\right\}$ from the best received signal $\max\left\{\left|Y_{n}\right|\right\}$ is expressed as follows:

(16) $$\begin{array}{c} \max_{\left[A_{1}\times A_{2}\right]}\left\{R_{\underset{n\in N}{n}-\underset{i=\left\{N,\ldots ,n\right\}}{x_{i}}}^{\left(T_{2}^{\left(odd\right)}\right)}\right\}=\frac{1}{2}\log_{2}\left(1+\max_{\left[A_{1}\times A_{2}\right]}\left\{\gamma_{\underset{n\in N}{\;n}-\underset{i=\left\{N,\ldots ,n\right\}}{x_{i}}}^{\left(T_{2}^{\left(odd\right)}\right)}\right\}\right). \end{array}$$

#### 2.2.2. Even Transmission Block

As with the first time slot in the odd transmission block, by applying the PS protocol, the first time slot in the even transmission block $T_{1}^{\left(even\right)}$ is used by the BS to transmit wireless energy and superimposed information simultaneously to coupled relays. The EH from the best channel in the pre-coding channel matrix at coupled relays $R_{1}$ and $R_{2}$ is expressed as follows:

(17) $$\begin{array}{c} E_{R_{1}}^{T_{1}^{\left(even\right)}}=\eta \lambda P_{S}\max_{\left[A_{0}\times A_{1}\right]}\left\{{\left|H_{0}\right|}^{2}\right\}, \end{array}$$

(18) $$\begin{array}{c} E_{R_{2}}^{T_{1}^{\left(even\right)}}=\eta \lambda P_{S}\max_{\left[A_{0}\times A_{1}\right]}\left\{{\left|G_{0}\right|}^{2}\right\}, \end{array}$$

where T1odd=ToddTodd22.

The BS broadcasts a superimposed signal by combining the independent messages $x_{i}$ of devices $D_{i}$ for $i=\left\{N,\ldots ,1\right\}$. Therefore, the received signal at coupled relays is expressed as follows:

(19) $$\begin{array}{c} \max_{\left[A_{0}\times A_{1}\right]}\left\{Y_{R_{1}}^{\left(T_{1}^{\left(even\right)}\right)}\right\}=\sqrt{1-\lambda }\max_{\left[A_{0}\times A_{1}\right]}\left\{\left|H_{0}\right|\right\}\sum_{i=N}^{1}\sqrt{\alpha_{i}P_{S}}x_{i}+n_{R_{1}}, \end{array}$$

(20) $$\begin{array}{c} \max_{\left[A_{0}\times A_{1}\right]}\left\{Y_{R_{2}}^{\left(T_{1}^{\left(even\right)}\right)}\right\}=\sqrt{1-\lambda }\max_{\left[A_{0}\times A_{1}\right]}\left\{\left|G_{0}\right|\right\}\sum_{i=N}^{1}\sqrt{\alpha_{i}P_{S}}x_{i}+n_{R_{2}}. \end{array}$$

In the second transmission block, SIC is implemented at the relay $R_{2}$. As with the SIC in the odd transmission block, the relay $R_{2}$ has to repeat SIC until it detect all data symbols $x_{i}$ for $i=\left\{N,\ldots ,1\right\}$ in the best received signal as (20). The SINRs are obtained when relay $R_{2}$ decodes the data symbols $x_{i}=\left\{x_{N},\ldots ,x_{1}\right\}$ as follows:

(21) $$\begin{array}{cc} \max_{\left[A_{0}\times A_{1}\right]}\left\{\underset{i=\left\{N,\ldots ,1\right\}}{\gamma_{R_{2}-x_{i}}^{\left(T_{1}^{\left(even\right)}\right)}}\right\} & =\frac{\left(1-\lambda \right)\max_{\left[A_{0}\times A_{1}\right]}\left\{{\left|G_{0}\right|}^{2}\right\}\alpha_{i}\rho_{S}}{\left(1-\lambda \right)\max_{\left[A_{0}\times A_{1}\right]}\left\{{\left|G_{0}\right|}^{2}\right\}\rho_{S}\sum_{j=1}^{i-1}\alpha_{j}+1},\;\mathrm{for}\;i>1, \end{array}$$

(22) $$\begin{array}{cc} \; & =\left(1-\lambda \right)\max_{\left[A_{0}\times A_{1}\right]}\left\{{\left|G_{0}\right|}^{2}\right\}\alpha_{i}\rho_{S},\;\;\mathrm{for}\;i=n=1. \end{array}$$

Similar to (8) and (8), maximization of the instantaneous bit-rate threshold achieved at the relay $R_{2}$ when the relay $R_{2}$ decodes data symbols $x_{i}$, where $i=\left\{N,\ldots ,1\right\}$ is expressed as follows:

(23) $$\begin{array}{c} \max_{\left[A_{0}\times A_{1}\right]}\left\{\underset{i=\left\{N,\ldots ,1\right\}}{R_{R_{2}-x_{i}}^{\left(T_{1}^{\left(even\right)}\right)}}\right\}=\frac{1}{2}\log_{2}\left(1+\max_{\left[A_{0}\times A_{1}\right]}\left\{\underset{i=\left\{N,\ldots ,1\right\}}{\gamma_{R_{2}-x_{i}}^{\left(T_{1}^{\left(even\right)}\right)}}\right\}\right). \end{array}$$

By applying a DF protocol, relay $R_{2}$ recovers the decoded data symbols $x_{i}$ for $i=\left\{N,\ldots ,1\right\}$ and forwards a beamforming superimposed signal to devices $D_{n}$ for $n=\left\{1,\ldots ,N\right\}$. The received signals in the second time slot $T_{2}^{\left(even\right)}$ in even transmission block $T^{\left(even\right)}$ at devices $D_{n}$ while EH at relay $R_{1}$ are expressed, respectively, as follows:

(24) $$\begin{array}{c} \max_{\left[A_{1}\times A_{2}\right]}\left\{Y_{n\in N}^{\left(T_{2}^{\left(even\right)}\right)}\right\}=\max_{\left[A_{1}\times A_{2}\right]}\left\{\left|G_{n}\right|\right\}\sum_{i=N}^{1}\sqrt{\alpha_{i}P_{R_{2}}}x_{i}+n_{n}, \end{array}$$

(25) $$\begin{array}{c} E_{R_{1}}^{\left(T_{2}^{\left(even\right)}\right)}=\eta \lambda P_{S}\max_{\left[A_{0}\times A_{1}\right]}\left\{{\left|H_{0}\right|}^{2}\right\}, \end{array}$$

where $P_{R_{2}}$ is the transmission power at relay $R_{2}$ and the pre-coding channel matrix $G_{n}$ is given by

(26) $$\begin{array}{c} \underset{n=\left\{1,\ldots ,N\right\}}{G_{n}}=\left[\begin{array}{ccc} g_{n}^{\left(1,1\right)} & \cdots & g_{n}^{\left(1,A_{2}\right)}\\ \vdots & \ddots & \vdots \\ g_{n}^{\left(A_{1},1\right)} & \cdots & g_{n}^{\left(A_{1},A_{2}\right)} \end{array}\right] \end{array}$$

where the channel $g_{n}^{\left(a_{1},a_{2}\right)}$ in the pre-coding matrix channel $G_{n}$, with $a_{1}\in A_{1}$ and $a_{2}\in A_{2}$, is a channel from transmitting antenna $a_{1}$ at relay $R_{2}$ to a receiving antenna $a_{2}$ at a device $D_{n}$, applying Rayleigh distributions for propagation. Each fading channel is represented by $g_{n}^{\left(.,.\right)}$ such that $g_{n}^{\left(.,.\right)}=v_{n}^{-\omega }$, where $v_{n}$ is the distance from relay $R_{2}$ to device $D_{n}$.

The SINRs obtained at devices $D_{n}$ for $n=\left\{1,\ldots ,N\right\}$ when they decode the data symbols $x_{i}$ for $i=\left\{N,\ldots ,n\right\}$ from the best received signal $\max_{\left[A_{1}\times A_{2}\right]}\left\{\left|Y_{n}^{\left(T_{2}^{\left(even\right)}\right)}\right|\right\}$ are expressed as follows:

(27) $$\begin{array}{cc} \max_{\left[A_{1}\times A_{2}\right]}\left\{\gamma_{\underset{n\in N}{\;n}-\underset{i=\left\{N,\ldots ,n\right\}}{x_{i}}}^{\left(T_{2}^{\left(even\right)}\right)}\right\} & =\frac{\max_{\left[A_{1}\times A_{2}\right]}\left\{{\left|G_{n}\right|}^{2}\right\}\alpha_{i}\rho_{R_{2}}}{\max_{\left[A_{1}\times A_{2}\right]}\left\{{\left|G_{n}\right|}^{2}\right\}\sum_{j=1}^{i-1}\alpha_{j}\rho_{R_{2}}+1},\;\mathrm{for}\;i>1, \end{array}$$

(28) $$\begin{array}{cc} \; & =\max_{\left[A_{1}\times A_{2}\right]}\left\{{\left|G_{n}\right|}^{2}\right\}\alpha_{i}\rho_{R_{2}},\;\;\mathrm{for}\;i=n=1, \end{array}$$

where SNR ρR2=PR2PR2N0N0.

Maximization of the achievable bit-rate thresholds achieved at devices $D_{n}$ when they decode the data symbols $x_{i}$ for $i=\left\{N,\ldots ,n\right\}$ from the best received signal $\max_{\left[A_{1}\times A_{2}\right]}\left\{\left|Y_{n}^{\left(T_{2}^{\left(even\right)}\right)}\right|\right\}$ is expressed as follows:

(29) $$\begin{array}{c} \max_{\left[A_{1}\times A_{2}\right]}\left\{R_{\underset{n\in N}{n}-\underset{i=\left\{N,\ldots ,n\right\}}{x_{i}}}^{\left(T_{2}^{\left(odd\right)}\right)}\right\}=\frac{1}{2}\log_{2}\left(1+\max_{\left[A_{1}\times A_{2}\right]}\left\{\gamma_{\underset{n\in N}{\;n}-\underset{i=\left\{N,\ldots ,n\right\}}{x_{i}}}^{\left(T_{2}^{\left(odd\right)}\right)}\right\}\right). \end{array}$$

## 3. System Performance Analysis

Many factors affect the system performance of wireless networks. The authors of [34] investigated the causes of OP at the BS, such as brief BS power supply variation, preventive BS activity state transition due to excessive temperature increase or decrease inside the BS rack, auto-recovery software and hardware failure, and temporal cell interference and congestion. The downlink MIMO-NOMA model with superposition transmission at BS and SIC at the terminal devices and the SIC processing is adopted in receiver side [35]. Therefore, our study considered OP at the receivers when the receivers could not successfully decode messages in the received signals. We analyze the system performance of the network model depicted in Figure 1 and delivers novel closed-forms of OP, system throughput and EE expressions at coupled relays $R=\left\{R_{1},R_{2}\right\}$ and devices $D_{n}$ for $n=\left\{1,\ldots ,N\right\}$.

### 3.1. Outage Probability at the Coupled Relays R

**Theorem** **1.** The outage event at a relay in the coupled relays R occurs when the relay cannot successfully decode at least a data symbol $x_{i}\in \left\{x_{N},\ldots ,x_{1}\right\}$ from the best received signal $\max_{\left[A_{1}\times A_{2}\right]}\left\{\left|Y_{R_{1}}^{\left(T_{2}^{\left(odd\right)}\right)}\right|\right\}$ for an odd transmission block or $\max_{\left[A_{1}\times A_{2}\right]}\left\{\left|Y_{R_{2}}^{\left(T_{2}^{\left(even\right)}\right)}\right|\right\}$ for an even transmission block, which is the best signal after TAS. Therefore, the coupled relays R receive the best superimposed signal $\max_{\left[A_{1}\times A_{2}\right]}\left\{\left|Y_{R}^{\left(T_{2}^{\left(\theta \right)}\right)}\right|\right\}$ by applying a TAS protocol to select the best signal from the pre-coding channel matrix $H_{0}$ for an odd transmission block or $G_{0}$ for an even transmission block to maximize the SINRs $\gamma_{R-x_{i}}^{\left(T_{1}^{\left(\theta \right)}\right)}$, as given by (8), (9), (21), and (21), and maximize the instantaneous bit-rate threshold $\max_{\left[A_{0}\times A_{1}\right]}\left\{\underset{i=\left\{N,\ldots ,1\right\}}{R_{R-x_{i}}^{\left(T_{1}^{\left(\theta \right)}\right)}}\right\}$, as given by (10) or (23). Maximization of the instantaneous bit rate threshold $\max_{\left[A_{0}\times A_{1}\right]}\left\{\underset{i=\left\{N,\ldots ,1\right\}}{R_{R-x_{i}}^{\left(T_{1}^{\left(\theta \right)}\right)}}\right\}$ is then compared to a device’s predefined bit-rate threshold $R_{i}^{*}$, where $i=\left\{N,\ldots ,1\right\}$. If maximization of the instantaneous bit-rate threshold $\max_{\left[A_{0}\times A_{1}\right]}\left\{\underset{i=\left\{N,\ldots ,1\right\}}{R_{R-x_{i}}^{\left(T_{1}^{\left(\theta \right)}\right)}}\right\}$ is less than a device’s predefined bit-rate threshold $R_{i}^{*}$, an outage event will occur, i.e, the OP at coupled relays R is expressed as follows:

(30) $$\begin{array}{cc} OP_{R}^{\left(T_{1}^{\left(\theta \right)}\right)} & =\mathrm{Pr}\left\{\max_{\left[A_{0}\times A_{1}\right]}\left\{R_{R-x_{N}}^{\left(T_{1}^{\left(\theta \right)}\right)}\right\}<R_{N}^{*}\right\}\\ \; & +\mathrm{Pr}\left\{\max_{\left[A_{0}\times A_{1}\right]}\left\{R_{R-x_{N}}^{\left(T_{1}^{\left(\theta \right)}\right)}\right\}\geq R_{N}^{*},\max_{\left[A_{0}\times A_{1}\right]}\left\{R_{R-x_{N-1}}^{\left(T_{1}^{\left(\theta \right)}\right)}\right\}<R_{N-1}^{*}\right\}\\ \; & +\\ \; & \vdots \\ \; & +\mathrm{Pr}\left\{\begin{array}{c} \max_{\left[A_{0}\times A_{1}\right]}\left\{R_{R-x_{N}}^{\left(T_{1}^{\left(\theta \right)}\right)}\right\}\geq R_{N}^{*},\max_{\left[A_{0}\times A_{1}\right]}\left\{R_{R-x_{N-1}}^{\left(T_{1}^{\left(\theta \right)}\right)}\right\}\geq R_{N-1}^{*},\ldots ,\\ \max_{\left[A_{0}\times A_{1}\right]}\left\{R_{R-x_{2}}^{\left(T_{1}^{\left(\theta \right)}\right)}\right\}\geq R_{2}^{*},\max_{\left[A_{0}\times A_{1}\right]}\left\{R_{R-x_{1}}^{\left(T_{1}^{\left(\theta \right)}\right)}\right\}<R_{1}^{*} \end{array}\right\} \end{array}$$

*We obtain the OP at coupled relays $R=\left\{R_{1},R_{2}\right\}$ in novel closed-form as follows:*

(31) $$\begin{array}{cc} OP_{R}^{\left(T_{1}^{\left(\theta \right)}\right)} & =\sum_{i=N}^{1}\left(\prod_{a_{0}=0}^{A_{0}}\prod_{a_{1}=1}^{A_{1}}\left(1-\exp\left(-\frac{\gamma_{i}^{*}}{\left(1-\lambda \right)\beta_{i}\rho_{S}\sigma_{R}^{2}}\right)\right)\right.\\ \; & \times \left.\underset{\mathrm{for}\;i<N}{\underset{︸}{\prod_{k=N}^{i+1}\left(1-\prod_{a_{0}=1}^{A_{1}}\prod_{a_{1}=1}^{A_{1}}\left(1-\exp\left(-\frac{\gamma_{k}^{*}}{\left(1-\lambda \right)\left(\alpha_{k}-\gamma_{k}^{*}\sum_{j=1}^{k-1}\alpha_{j}\right)\rho_{S}\sigma_{R}^{2}}\right)\right)\right)}}\right), \end{array}$$

*where $\beta_{i}$ is given by*

(32) $$\begin{array}{cc} \beta_{i} & =\alpha_{i}-\gamma_{i}^{*}\sum_{j=1}^{i-1}\alpha_{j},\;\;for\;i>1, \end{array}$$

(33) $$\begin{array}{cc} \beta_{i} & =\alpha_{i},\;\;\;\;\;for\;i=1. \end{array}$$

See Appendix A for proof.

**Remark** **1.** *If the network has a large number of devices, it is challenge to apply the expression (30) in Monte Carlo simulations. Fortunately, the authors of [12] analyzed a network model with multiple relays and multiple devices. The authors presented the expressions for OP at the relays as [12] (Equation (33)). It is important to mention that the present paper extends the work of the previous study [12] by deploying massive MIMO and TAS techniques. From [12] (Equation (33)), the OP expression at the coupled relay R in (30) can be rewritten as follows:*

(34) $$\begin{array}{c} OP_{R}^{\left(T_{1}^{\left(\theta \right)}\right)}\;=1-\prod_{i=N}^{1}\underset{Q_{i}}{\underset{︸}{\mathrm{Pr}\left\{\max\left\{R_{R-x_{i}}^{\left(T_{1}^{\left(\theta \right)}\right)}\right\}\geq R_{i}^{*}\right\}}}. \end{array}$$

*From (34), we obtain a novel expression of OP at the coupled relay R in closed-form by applying the Cumulative Density Function (CDF) as defined in [13] (Equation (71)):*

(35) $$\begin{array}{cc} \; & OP_{R}^{\left(T_{1}^{\left(\theta \right)}\right)}=1-\prod_{i=N}^{1}\left(1-\sum_{\psi =0}^{A_{0}A_{1}}\frac{{\left(-1\right)}^{\psi }\left(A_{0}A_{1}\right)!}{\psi !\left(A_{0}A_{1}-\psi \right)!}\exp\left(-\frac{\psi \gamma_{i}^{*}}{\left(1-\lambda \right)\beta_{i}\rho_{S}\sigma_{R}^{2}}\right)\right). \end{array}$$

See Appendix B for proof.

### 3.2. Outage Probability at Devices

**Theorem** **2.** *The outage event in an odd or even transmission block at devices $D_{n}$ for $n=\left\{1,\ldots ,N\right\}$ occurs when, on the one hand, the relays $R_{1}$ and $R_{2}$ for odd and even transmission blocks, respectively, cannot successfully decode at least data symbol $x_{i}$ for $i=\left\{N,\ldots ,n\right\}$ from the best received signal $\max_{\left[A_{0}\times A_{1}\right]}\left\{\left|Y_{R_{1}}^{\left(T_{1}^{\left(odd\right)}\right)}\right|\right\}$ as (5) or $\max_{\left[A_{0}\times A_{1}\right]}\left\{\left|Y_{R_{2}}^{\left(T_{1}^{\left(even\right)}\right)}\right|\right\}$ as (20). On the other hand, the coupled relays can successfully decode all data symbol $x_{i}$ for $i=\left\{N,\ldots ,n\right\}$, but device $D_{n}$ cannot successfully decode at least data symbols $x_{i}$ for $i=\left\{N,\ldots ,n\right\}$ from the best received signal $\max_{\left[A_{1}\times A_{2}\right]}\left\{\left|Y_{n}^{\left(T_{2}^{\left(odd\right)}\right)}\right|\right\}$ as (11) for an odd transmission block or $\max_{\left[A_{1}\times A_{2}\right]}\left\{\left|Y_{n}^{\left(T_{2}^{\left(even\right)}\right)}\right|\right\}$ as (24) for an even transmission block.*

*Therefore, the OP at devices in an odd or even transmission block is expressed as follows:*

(36) $$\begin{array}{cc} OP_{n}^{\left(\theta \right)} & =\sum_{i=N}^{n}\left\{\mathrm{Pr}\left\{\max_{\left[A_{0}\times A_{1}\right]}\left\{R_{R-x_{i}}^{\left(T_{1}^{\left(\theta \right)}\right)}\right\}<R_{i}^{*}\right\}\underset{\mathrm{for}\;i<N}{\underset{︸}{\prod_{k=N}^{i+1}\mathrm{Pr}\left\{\max_{\left[A_{0}\times A_{1}\right]}\left\{R_{R-x_{k}}^{\left(T_{1}^{\left(\theta \right)}\right)}\right\}\geq R_{k}^{*}\right\}}}\right\}\\ \; & +\sum_{i=N}^{n}\left\{\mathrm{Pr}\left\{\max_{\left[A_{1}\times A_{2}\right]}\left\{R_{n-x_{i}}^{\left(T_{2}^{\left(\theta \right)}\right)}\right\}<R_{i}^{*}\right\}\underset{\mathrm{for}\;i<N}{\underset{︸}{\prod_{k=N}^{i+1}\mathrm{Pr}\left\{\max_{\left[A_{1}\times A_{2}\right]}\left\{R_{n-x_{k}}^{\left(T_{2}^{\left(\theta \right)}\right)}\right\}\geq R_{k}^{*}\right\}}}\right.\\ \; & \;\;\;\;\;\;\;\;\times \left.\prod_{t=N}^{n}\mathrm{Pr}\left\{\max_{\left[A_{0}\times A_{1}\right]}\left\{R_{R-x_{t}}^{\left(T_{1}^{\left(\theta \right)}\right)}\right\}\geq R_{t}^{*}\right\}\right\} \end{array}$$

*From (36), the OP at user $D_{n}$ is obtained in closed-form as follows:*

(37) $$\begin{array}{cc} OP_{n\in N}^{\left(\theta \right)} & =\sum_{i=N}^{n}\left(\underset{Q_{i}}{\underset{︸}{\prod_{a_{0}}^{A_{0}}\prod_{a_{1}}^{A_{1}}\left(1-\exp\left(-\frac{\gamma_{i}^{*}}{\left(1-\lambda \right)\beta_{i}\rho_{S}\sigma_{R}^{2}}\right)\right)}}\underset{\mathrm{for}\;i<N}{\underset{︸}{\prod_{k=N}^{i+1}\left(1-Q_{k}\right)}}\right)\\ \; & +\sum_{i=N}^{n}\left(\underset{K_{i}}{\underset{︸}{\prod_{a_{1}}^{A_{1}}\prod_{a_{2}}^{A_{2}}\left(1-\exp\left(-\frac{\gamma_{i}^{*}}{\beta_{i}\rho_{R}\sigma_{n}^{2}}\right)\right)}}\underset{\mathrm{for}\;i<N}{\underset{︸}{\prod_{k=N}^{i+1}\left(1-K_{k}\right)}}\prod_{t=N}^{n}\left(1-Q_{t}\right)\right) \end{array}$$

*where $\sigma_{n}^{2}=E\left\{{\left|H_{n}\right|}^{2}\right\}$. Note that θ=odd and $R=R_{1}$, or θ=even and $R=R_{2}$.*

See Appendix C for proof.

Similar to (30), note that Expression (36) is challenging in Monte Carlo simulations if the network has a large number of devices. Therefore, the OP at devices $D_{n}$ can be rewritten as follows:

(38) $$\begin{array}{c} OP_{n}^{\left(\theta \right)}=1-\prod_{i=N}^{n}\mathrm{Pr}\left\{\max_{\left[A_{0}\times A_{1}\right]}\left\{R_{R-x_{i}}^{\left(T_{1}^{\left(\theta \right)}\right)}\right\}\geq R_{i},\max_{\left[A_{1}\times A_{2}\right]}\left\{R_{n-x_{i}}^{\left(T_{2}^{\left(\theta \right)}\right)}\right\}\geq R_{i}\right\}. \end{array}$$

From (38), we obtain the expression of OP at devices $D_{n}$ in closed-form as follows:

(39) $$\begin{array}{cc} OP_{n}^{\left(\theta \right)} & =1-\prod_{i=N}^{n}\left(\left(1-\sum_{\psi =0}^{A_{0}A_{1}}\frac{{\left(-1\right)}^{\psi }\left(A_{0}A_{1}\right)!}{\psi !\left(A_{0}A_{1}-\psi \right)!}\exp\left(-\frac{\psi \gamma_{i}^{*}}{\left(1-\lambda \right)\beta_{i}\rho_{S}\sigma_{R}^{2}}\right)\right)\right.\\ \; & \;\;\;\times \left.\left(1-\sum_{\mu =0}^{A_{1}A_{2}}\frac{{\left(-1\right)}^{\mu }\left(A_{1}A_{2}\right)!}{\mu !\left(A_{1}A_{2}-\mu \right)!}\exp\left(-\frac{\mu \gamma_{i}^{*}}{\beta_{i}\rho_{R}\sigma_{n}^{2}}\right)\right)\right). \end{array}$$

See Appendix D for the proof.

### 3.3. System Throughput

In Figure 1 and Figure 2, the individual system model has two transmission blocks. Each odd or even transmission block is separated into two time slots. The achievable system throughput in an odd or even transmission block is the sum of the minimal device throughput at the relay and device in the same transmission block. Therefore, the system throughput is expressed as follows:

(40) $$\begin{array}{c} TP_{\;}^{\left(\theta \right)}=\sum_{n=1}^{N}\left(1-\max\left\{OP_{R-x_{n}}^{\left(T_{1}^{\left(\theta \right)}\right)},OP_{n-x_{n}}^{\left(T_{2}^{\left(\theta \right)}\right)}\right\}\right)R_{n}^{*} \end{array}$$

### 3.4. Energy Efficiency

Green wireless networks require a higher throughput yet use lower energy. To achieve this aim, the present study deploys a massive MIMO technique and SWIPT protocol. As a result, the EE performance indicates the sum of device throughput and sum of transmission power at the BS and coupled relay ratio in the same transmission block. Therefore, the EE performance of an individual network model as given by Figure 1 is expressed as follows:

(41) $$\begin{array}{c} EE_{\;}^{\left(\theta \right)}=\frac{\sum_{n=1}^{N}\left(1-\max\left\{OP_{R-x_{n}}^{\left(T_{1}^{\left(\theta \right)}\right)},OP_{n-x_{n}}^{\left(T_{2}^{\left(\theta \right)}\right)}\right\}\right)R_{n}^{*}}{\left(1-\lambda \right)\rho_{S}+\rho_{R}}. \end{array}$$

## 4. Numerical Results and Discussion

To investigate the system performance of the massive MIMO-NOMA network model shown in Figure 1, we propose parameters for both a theoretical analysis and the Monte Carlo simulations, as shown in Table 1.

Let a wireless network contain coupled relays and three devices (N=3). The coupled relays are allocated nearby. The distances from the BS to the coupled relays are $d_{R_{1}}=d_{R_{2}}=10$ m, and the distances from the coupled relays to devices $D_{1}$, $D_{2}$ and $D_{3}$ are $d_{1}=v_{1}=5$ m, $d_{2}=v_{2}=7$ m and $d_{3}=v_{3}=10$ m, respectively. The path-loss exponent refers to an indoor environment with λ=4. By modeling the more challenging indoor environment, the performance bound of the less challenging outdoor scenario is therefore also covered. In 4G Long-Term Evolution (LTE) release 8, the maximum numbers of antennas at the BS and user equipment are 4T4R and 1T2R, respectively. In 4G LTE-Advanced (LTE-A), the number of antennas is greater to allow the use of 8T8R at BSs and 2T2R at devices in LTE-A schemes. Therefore, massive MIMO networks need at least an 8T8R antenna array at the BS. However, the present study assumes a certain number of antennas equipped at the BS ($A_{0}=4$), coupled relays $R_{1}$ ($A_{1}=4$), $R_{2}$ ($A_{1}=16$), and devices ($A_{2}=2$) to prove the the benefits of massive MIMO (even transmission block) over MIMO (odd transmission block). The fading channel from the transmitting antennas at the BS to the receiving antennas at the coupled relays are distributed over Rayleigh fading channels. Based on distances and the path-loss exponent, the expected channel gains from the BS to coupled relays are $\sigma_{R_{1}}^{2}=\sigma_{R_{2}}^{2}=1\times 10^{-4}$ and from the coupled relays to devices $D_{1}$, $D_{2}$, and $D_{3}$ are $\sigma_{1}^{2}=16\times 10^{-4}$, $\sigma_{2}^{2}=4.1649\times 10^{-4}$, and $\sigma_{3}^{2}=1\times 10^{-4}$. Each fading channel randomly generates 1e6 experiments. To simplify, the devices require the same bit-rate thresholds $R_{1}^{*}=R_{2}^{*}=R_{3}^{*}=0.1bps/Hz$ and SNR $\rho_{S}=\rho_{R}=\left\{0,\ldots ,30\right\}$ dB. By applying (7), the PA factors for devices $D_{1}$, $D_{2}$, and $D_{3}$ are $\alpha_{1}=0.1667$, $\alpha_{2}=0.3333$, and $\alpha_{3}=0.5$, respectively. The present study assumes that coupled relays may fully collect EH (η=1). The PS factor in an odd transmission block is λ=0.4. Therefore, $0.4P_{S}$ and $0.6P_{S}$ are applied to EH and DT processing, respectively, whereas the PS factor in an even transmission block is reduced (λ=0.4) since the relay $R_{2}$ in this block is equipped with a greater number of antennas than relay $R_{1}$ in an odd transmission block.

Figure 3 plots the OP performance at relay $R_{1}$ and devices in odd transmission blocks. Note the various markers and line plot analysis (Ana) and simulation (Sim) results. The analysis results of OP performance at relay $R_{1}$ are given by (30) or (34) and for devices $D_{n}$ by (36) or (38). The analysis results were verified with Monte Carlo simulations for relay $R_{1}$ given by (31) or (35) and for devices by (37) or (39), where $R=R_{1}$ and θ=odd. Figure 3 illustrates that device $D_{3}$ achieved the best OP results, even though device $D_{3}$ was the farthest device and therefore allocated the biggest PA factor $\alpha_{3}=0.5$. When SRN ρ→∞, the OP results of relay $R_{1}$ and devices tend toward zero.

**Figure 4:**
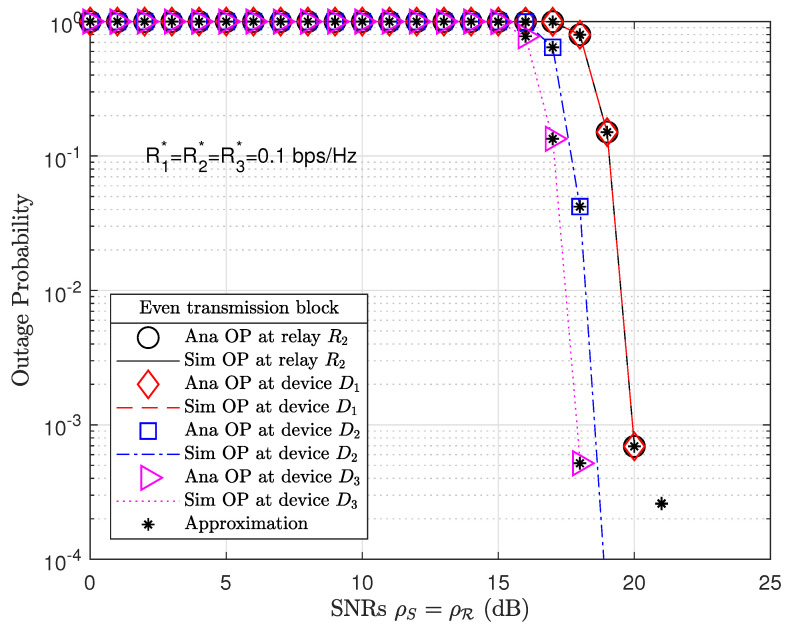
OP at relay $R_{2}$ and devices $D_{n}$ for $n=\left\{1,\ldots ,N\right\}$ in an even transmission block, where the number of antennas equipped at the BS, relay $R_{2}$ and devices $D_{n}$ are $A_{0}=4$, $A_{1}=16$, and $A_{2}=2$, respectively, and the PS factor λ=0.6.

Figure 4 plots the OP performance at relay $R_{2}$ and devices in even transmission blocks. To improve the networking capacity and energy, we equipped a large number of antennas at relay $R_{2}$ and increased the PS factor λ=0.6. By increasing the PS factor, relay $R_{1}$ was able to harvest more energy but relay $R_{2}$ could receive weak signals. However, we may observe that the OP performance at relay $R_{2}$ and devices in even transmission blocks (Figure 4) achieved better results than OP performance at relay $R_{1}$ and devices in odd transmission blocks (Figure 3). The analysis results of OP performance for relay $R_{2}$ are given by (30) or (34) and for devices $D_{n}$ by (36) or (38), where $R=R_{2}$ and θ=even. The analysis results were verified with Monte Carlo simulations for relay $R_{2}$ given by (31) or (35) and for devices by (37) or (39), where $R=R_{2}$ and θ=even.

We may observe that the OP performances at relay $R_{2}$ and devices in even transmission blocks outperform those at relay $R_{1}$ and devices in odd transmission blocks at high SNRs such as $\rho_{S}=\rho_{R}=20$ dB. The present study thus exploits the advantages of a massive MIMO-NOMA network compared to a MIMO-NOMA network. We equipped a greater number of antennas on relay $R_{2}$ ($A_{1}=16$) than on relay $R_{1}$ ($A_{1}=4$). As a result, we obtained the respective pre-coding channel matrix sizes of $\left[4\times 16\right]$ and $\left[16\times 2\right]$ for $G_{0}$ and $G_{n}$, which, in the even transmission blocks, were much larger than the pre-coding channel matrix sizes of $\left[4\times 4\right]$ and $\left[4\times 2\right]$ for $H_{0}$ and $H_{n}$ in odd transmission blocks. From Expressions (8), (9), (14), (15), (21), (22), (27), and (28) and by applying the TAS protocol, only the best channels from the pre-coding channel matrices are selected for data decoding. Therefore, the relay $R_{2}$ and devices in even transmission blocks have better OP performance than relay $R_{1}$ and devices in odd transmission blocks under the same simulation parameters.

**Figure 5:**
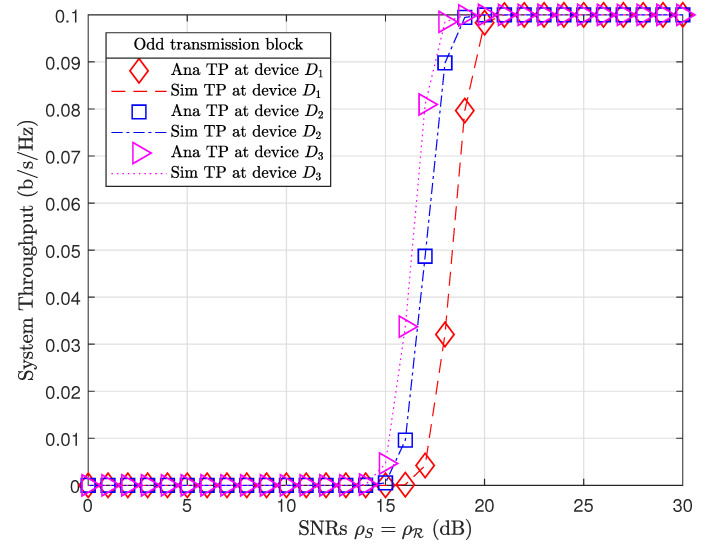
System throughput in odd transmission blocks.

**Figure 6:**
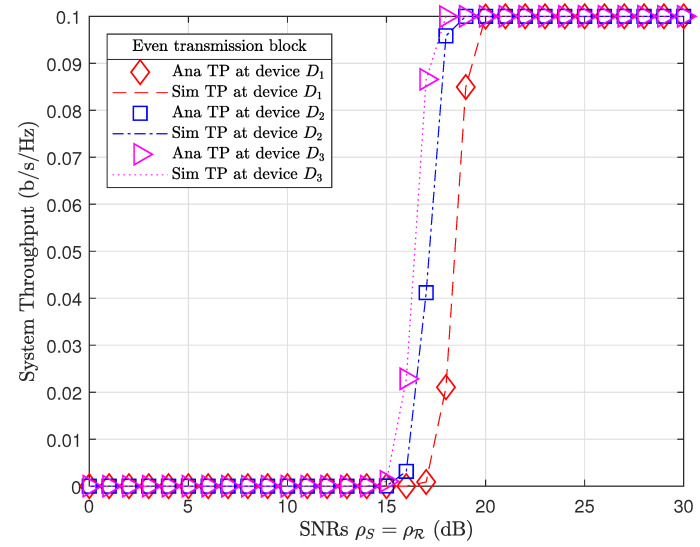
System throughput in even transmission blocks.

Figure 5 and Figure 6 plot the system throughput performance at devices in odd and even transmission blocks, respectively. Even though device $D_{3}$ has the farthest distance from coupled relays ($d_{3}=v_{3}=10$ m), device $D_{3}$ always achieved the best throughput performance compared to other devices. It is interesting that the system throughput results of relay $R_{1}$ and devices in odd transmission blocks (Figure 5) are similar to the system throughput results of relay $R_{2}$ and devices in even transmission blocks (Figure 6) at the same SNR.

To illustrate, we extract the investigated results from Matlab software. At a SNR range $\rho_{S}=\rho_{R}=\left\{15,\ldots ,21\right\}\;dB$, device throughput in odd and even transmission blocks achieved $TP_{\;}^{\left(odd\right)}=\left\{0.0051,0.0435,0.1338,0.2204,0.279,0.2983,0.3\right\}$ and $TP_{\;}^{\left(even\right)}=\left\{0.0011,0.0261,0.1287,0.2169,0.2849,0.2999,0.3\right\}$, respectively. We may observe that device throughput in odd transmission blocks outperformed device throughput in even transmission blocks at low SNRs, i.e., $\rho_{S}=\rho_{R}=\left\{15,\ldots ,18\right\}\;dB$. However, device throughput in even transmission blocks improved and outperformed device throughput in odd transmission blocks at high SNRs, i.e., $\rho_{S}=\rho_{R}=\left\{19,20\right\}$ dB. Device throughput also tended toward their data rate thresholds, i.e., $R_{1}^{*}=R_{2}^{*}=R_{3}^{*}=0.1\;b/s/Hz$ when the SNRs $\rho_{S}=\rho_{R}\to \infty$. As a result, system throughput in both odd and even transmission blocks $TP^{\left(odd\right)}=TP^{\left(even\right)}=R_{1}^{*}+R_{2}^{*}+R_{3}^{*}=0.3\;b/s/Hz$ at high SNRs $\rho_{S}=\rho_{R}\geq 20$ dB. It is important to note that the PS factor in even transmission blocks (λ=0.6) was greater than the PS factor in odd transmission blocks (λ=0.4). Therefore, the relay $R_{1}$ in even transmission blocks harvested more energy than relay $R_{2}$ in odd transmission blocks. Certainly, even transmission blocks achieved better EE performance than odd transmission blocks.

**Figure 7:**
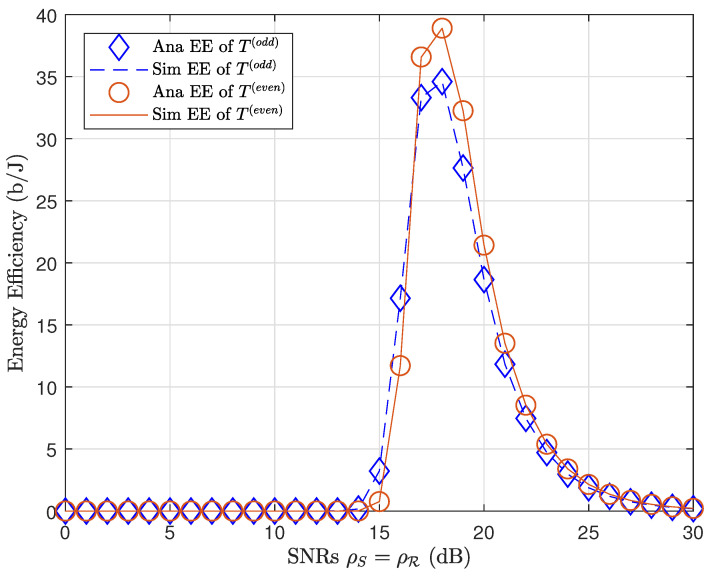
EE of a MIMO network (odd transmission block) compared to a massive MIMO network (even transmission block).

Figure 7 plot the EE performance of odd even transmission blocks. We may observe that EE performance in the even transmission block with massive MIMO had a higher peak than the odd transmission block. The massive MIMO technique therefore not only provided greater throughput but also consumed less energy.

## 5. Conclusions

This paper proposes a design for a switchable coupled relay model to assist a massive MIMO-NOMA wireless network in serving multiple devices and extending a network’s lifespan. A diagram of two transmission blocks illustrates signal propagation and EH processing. Propagation and formulations are analyzed. We derive the closed-form novel expressions for OP at the coupled relays and devices. The theoretical results show that the massive MIMO technique in combination with TAS and SWIPT protocols in an underlay cooperative NOMA network provides higher throughput and consumes lower energy. The obtained results verify the massive MIMO technique as effective for 5G wireless networks. The present paper offers the potential for the practical application of a massive MIMO-NOMA network model assisted by switchable coupled relays, for example in a water environment where relays and devices are barely powered. Our massive MIMO-NOMA assisted by switchable coupled relays in combination with TAS and EH protocols can not only improve OP and system throughput performance but also extend the network’s lifespan. Specifically, EH at the relay may be used to forward signals without consuming the relay’s own energy. This is promising as a potential solution in extending a network’s lifespan.

## Figures and Tables

**Table 1:** Table of parameters.

Variables	Values	Units
N	3	
$d_{R_{1}}=d_{R_{2}}$	10	metres
$d_{1}=v_{1}$	5	metres
$d_{2}=v_{2}$	7	metres
$d_{3}=v_{3}$	10	metres
$\alpha_{1}$	0.1667	
$\alpha_{2}$	0.3333	
$\alpha_{3}$	0.5	
ω	4	
$\sigma_{R_{1}}^{2}=\sigma_{R_{2}}^{2}$	$1\times 10^{-4}$	
$\sigma_{1}^{2}$	$16\times 10^{-4}$
$\sigma_{2}^{2}$	$4.1649\times 10^{-4}$
$\sigma_{3}^{2}$	$1\times 10^{-4}$
$R_{1}^{*}=R_{2}^{*}=R_{3}^{*}$	0.1	bps/Hz
$\rho_{S}=\rho_{R_{1}}=\rho_{R_{2}}$	$\left\{0,\ldots ,30\right\}$	dB
η	1	
$A_{0}$	4	
$A_{2}$	2	
Odd transmission block		
$A_{1}$	4	
λ	0.4	
Even transmission block		
$A_{1}$	16	
λ	0.6	

## Data Availability

Data was randomly generated when we were running program. There is no data to share.

## Abbreviations

The following abbreviations are used in this manuscript:

5G	Fifth-Generation
AF	Amplify-and-Forward
AWGN	Adaptive White Gaussian Noise
BS	Base Station
CSI	Channel State Information
DF	Decode-and-Forward
EE	Energy Efficiency
EH	Energy Harvesting
FD	Full-Duplex
HD	Half-Duplex
IoTs	Internet of Things
MIMO	Multi-Input-Multi-Output
NOMA	Non-Orthogonal Multiple Access
OP	Outage Probability
PA	Power Allocation
PS	Power Splitting
QoS	Quality of Service
RF	Radio Frequency
SIC	Successive Interference Cancellation
SINR	Signal-to-Interference-plus-Noise Ratio
SISO	Single-Input-Single-Output
SNR	Signal-to-Noise Ratio
SWIPT	Simultaneously Wireless Information Power Transmit
TAS	Transmit Antennas Selection
TS	Time Switching
WIP	Wireless Information Power

## Notations

The following notations are used in this manuscript:

*N*	N≥1	Number of devices
$R_{1}$, $R_{2}$		Coupled relays
$d_{R_{1}}$, $d_{R_{2}}$		Distances from BS to relays
R	$R=\left\{R_{1},R_{2}\right\}$	Set of relays
$D_{n}$	n∈N	Devices
$d_{n}$	n∈N	Distances from relay $R_{1}$ to devices
$v_{n}$	n∈N	Distances from relay $R_{2}$ to devices
$A_{0}$, $A_{1}$, $A_{2}$	$A_{0}>1$, $A_{1}>1$, $A_{2}>1$	Number of antennas on BS, $R_{1}$ and $R_{2}$, respectively
ω		Path-loss exponent factor
$H_{0}$, $G_{0}$		Pre-coding channel matrices from BS to $R_{1}$ and $R_{2}$
$h_{0}^{\left(a_{0},a_{1}\right)}$, $g_{0}^{\left(a_{0},a_{1}\right)}$	$a_{0}\in A_{0}$, $a_{1}\in A_{1}$	Channels from BS to $R_{1}$ and $R_{2}$
$H_{n}$, $G_{n}$		Pre-coding channel matrices from $R_{1}$ and $R_{2}$ to devices
θ	$\theta =\left\{odd,even\right\}$	binary value
$T^{\left(\theta \right)}$	$\theta =\left\{odd,even\right\}$	Odd/even transmission block
$h_{n}^{\left(a_{0},a_{1}\right)}$, $g_{n}^{\left(a_{0},a_{1}\right)}$	n∈N,$a_{0}\in A_{0}$, $a_{1}\in A_{1}$	Channels from relays $R_{1}$ and $R_{2}$ to devices
$T_{1}^{\left(\theta \right)}$,$T_{2}^{\left(\theta \right)}$		First/second time slot in odd/even transmission block
λ	0≤λ≤1	Power splitting factor
η	0≤η≤1	Collect factor
$P_{S}$,$P_{R}$		Power domain on BS and relays
$\rho_{S}$,$\rho_{R}$		SNR on BS and relays
$\alpha_{i}$	i∈N, $\sum_{i}\alpha_{i}=1$, $\alpha_{1}<\ldots <\alpha_{N}$	PA factors for devices
$x_{i}$	i∈N	Data of devices
$n_{{\;}_{R}}$, $n_{n}$,	n∈N	AWGN at relays and devices
$Y_{R}^{\left(T_{1}^{\left(\theta \right)}\right)}$	$\theta =\left\{odd,even\right\}$, $R=\left\{R_{1},R_{2}\right\}$	Received signals at relays
$Y_{n}^{\left(T_{2}^{\left(\theta \right)}\right)}$	$\theta =\left\{odd,even\right\}$, n∈N	Received signals at devices
$R_{i}^{*}$	i∈N	Devices’ data rate thresholds
$\gamma_{i}^{*}$	i∈N	SINR thresholds
$\gamma_{R-x_{i}}^{\left(T_{1}^{\left(\theta \right)}\right)}$	$\theta =\left\{odd,even\right\}$, $R=\left\{R_{1},R_{2}\right\}$, i∈N	SINRs at relays decode symbol $x_{i}$
$\gamma_{n-x_{i}}^{\left(T_{2}^{\left(\theta \right)}\right)}$	$\theta =\left\{odd,even\right\}$, n∈N, i∈n	SINRs at devices decode symbol $x_{i}$
$R_{R-x_{i}}^{\left(T_{1}^{\left(\theta \right)}\right)}$	$\theta =\left\{odd,even\right\}$, $R=\left\{R_{1},R_{2}\right\}$, i∈N	Relays’ instantaneous bit-rate thresholds
$R_{n-x_{i}}^{\left(T_{2}^{\left(\theta \right)}\right)}$	$\theta =\left\{odd,even\right\}$, n∈N, i∈n	Devices’ instantaneous bit-rate thresholds
$E_{R}^{\left(T_{1}^{\left(\theta \right)}\right)}$	$\theta =\left\{odd,even\right\}$, $R=\left\{R_{1},R_{2}\right\}$	EH at relay $R_{1}$ or $R_{2}$ in odd/even transmission block
$OP_{R}^{\left(T_{1}^{\left(\theta \right)}\right)}$	$\theta =\left\{odd,even\right\}$, $R=\left\{R_{1},R_{2}\right\}$	OP at relays
$OP_{n}^{\left(\theta \right)}$	$\theta =\left\{odd,even\right\}$, n∈N	OP at devices
$TP_{\;}^{\left(\theta \right)}$	$\theta =\left\{odd,even\right\}$	System throughput in odd/even transmission block
$EE_{\;}^{\left(\theta \right)}$	$\theta =\left\{odd,even\right\}$	Energy efficiency in odd/even transmission block

## Appendix A. Proof of Theorem 1

From (30), let

(A1) $$\begin{array}{c} \underset{i=\left\{N,\ldots ,1\right\}}{L_{R-x_{i}}^{\left(T_{1}^{\left(\theta \right)}\right)}}=\mathrm{Pr}\left\{\max_{\left[A_{0}\times A_{1}\right]}\left\{R_{R-x_{i}}^{\left(T_{1}^{\left(\theta \right)}\right)}\right\}<R_{i}^{*}\right\}\underset{\mathrm{for}\;i<N}{\underset{︸}{\prod_{k=N}^{i+1}\mathrm{Pr}\left\{\max_{\left[A_{0}\times A_{1}\right]}\left\{R_{R-x_{i}}^{\left(T_{1}^{\left(\theta \right)}\right)}\right\}\geq R_{i}^{*}\right\}}} \end{array}$$

After some algebraic manipulation, we obtain

(A2) $$\begin{array}{c} \underset{i=\left\{N,\ldots ,1\right\}}{L_{R-x_{i}}^{\left(T_{1}^{\left(\theta \right)}\right)}}=\mathrm{Pr}\left\{\max_{\left[A_{0}\times A_{1}\right]}\left\{{\left|H_{0}\right|}^{2}\right\}<\frac{\gamma_{i}^{*}}{\left(1-\lambda \right)\beta_{i}\rho_{S}}\right\}\underset{\mathrm{for}\;i<N}{\underset{︸}{\prod_{k=N}^{i+1}\mathrm{Pr}\left\{\max_{\left[A_{0}\times A_{1}\right]}\left\{{\left|H_{0}\right|}^{2}\right\}\geq \frac{\gamma_{i}^{*}}{\left(1-\lambda \right)\beta_{i}\rho_{S}}\right\}}}. \end{array}$$

From PDF expressions [23] (Equations (59) and (60)), we obtain

(A3) LR−xiT1θi=N,…,1=∏a0=1A0∏a1=1A1∫0γi*1−λβiρSexp−xxσR2σR2σR2dx∏k=Ni+1∏a0A0∏a1A1∫γk*1−λβkρS∞exp−yyσR2σR2σR2dy︸i<N=∏a0=1A0∏a1=1A11−exp−γi*1−λβiρSσR2∏k=Ni+11−∏a0A0∏a1A11−exp−γk*1−λβkρSσR2︸i<N.

By substituting (A3) into (30), we obtain the expression of OP at coupled relays R, as shown in (31).

From (34), we obtain

(A4) $$\begin{array}{c} Q_{i}=\mathrm{Pr}\left\{\max_{\left[A_{0}\times A_{1}\right]}\left\{R_{R-x_{i}}^{\left(T_{1}^{\left(\theta \right)}\right)}\right\}\geq R_{i}^{*}\right\}=\mathrm{Pr}\left\{\max_{\left[A_{0}\times A_{1}\right]}\left\{\gamma_{R-x_{i}}^{\left(T_{1}^{\left(\theta \right)}\right)}\right\}\geq \gamma_{i}^{*}\right\}. \end{array}$$

## Appendix B. Proof of Remark 1

From [23] (Equation (71)), we obtain the CDF expression as follows:

(A5) $$\begin{array}{cc} \; & F_{\max_{\left[A_{0}\times A_{1}\right]}\left\{\gamma_{R-x_{i}}^{\left(T_{1}^{\left(\theta \right)}\right)}\right\}}\left(\gamma \right)=\sum_{\psi =0}^{A_{0}A_{1}}\frac{{\left(-1\right)}^{\psi }\left(A_{0}A_{1}\right)!}{\psi !\left(A_{0}A_{1}-\psi \right)!}\exp\left(-\frac{\psi \gamma }{\left(1-\lambda \right)\left(\alpha_{i}-\gamma \sum_{j=1}^{i-1}\alpha_{j}\right)\rho_{S}\sigma_{R}^{2}}\right), \end{array}$$

(A6) $$\begin{array}{cc} \; & F_{\max_{\left[A_{0}\times A_{1}\right]}\left\{\gamma_{R-x_{i}}^{\left(T_{1}^{\left(\theta \right)}\right)}\right\}}\left(\gamma \right)=\sum_{\psi =0}^{A_{0}A_{1}}\frac{{\left(-1\right)}^{\psi }\left(A_{0}A_{1}\right)!}{\psi !\left(A_{0}A_{1}-\psi \right)!}\exp\left(-\frac{\psi \gamma }{\left(1-\lambda \right)\alpha_{i}\rho_{S}\sigma_{R}^{2}}\right), \end{array}$$

where Expression (A5) is with i>1 and γ<αiαi∑j=1i−1αj∑j=1i−1αj. If γ≥αiαi∑j=1i−1αj∑j=1i−1αj, Expression (A5) refers to one. Expression (A6) is with i=1.

By applying the CDF expressions as (A5) and (A6), we obtain

(A7) $$\begin{array}{cc} Q_{i} & =1-F_{\max_{\left[A_{0}\times A_{1}\right]}\left\{\gamma_{R-x_{i}}^{\left(T_{1}^{\left(\theta \right)}\right)}\right\}}\left(\gamma_{i}^{*}\right)=1-\sum_{\psi =0}^{A_{0}A_{1}}\frac{{\left(-1\right)}^{\psi }\left(A_{0}A_{1}\right)!}{\psi !\left(A_{0}A_{1}-\psi \right)!}\exp\left(-\frac{\psi \gamma_{i}^{*}}{\left(1-\lambda \right)\beta_{i}\rho_{S}\sigma_{R}^{2}}\right). \end{array}$$

By substituting (A7) into (34), we obtain OP at coupled relay, as shown in (35).

## Appendix C. Proof of Theorem 2

From (36), we obtain

(A8) OPnθ=∑i=Nn∏a0A0∏a1A1∫0γi*1−λβiρSexp−x−xσR2σR2σR2dx∏k=Ni+1∏a0A0∏a1A1∫γk*1−λβkρS∞exp−x−xσR2σR2σR2dx+∑i=Nn∏a1A1∏a2A2∫0γi*βiρRexp−x−xσn2σn2σn2dx∏k=Ni+1∏a1A1∏a2A2∫γk*βkρR∞exp−x−xσn2σn2σn2dx×∏t=Nn∏a0A0∏a1A1∫γt*1−λβtρS∞exp−x−xσR2σR2σR2dx

Expression (A8) can be solved and obtained in closed-form, as shown in (37).

## Appendix D. Proof of Remark 2

From (38), we obtain

(A9) $$\begin{array}{c} OP_{n}^{\left(\theta \right)}=1-\prod_{i=N}^{n}\left(1-F_{\max_{\left[A_{0}\times A_{1}\right]}\left\{\gamma_{R-x_{i}}^{\left(T_{1}^{\left(\theta \right)}\right)}\right\}}\left(\gamma_{i}^{*}\right)\right)\left(1-F_{\max_{\left[A_{1}\times A_{2}\right]}\left\{\gamma_{n-x_{i}}^{\left(T_{2}^{\left(\theta \right)}\right)}\right\}}\left(\gamma_{i}^{*}\right)\right), \end{array}$$

where $F_{\max_{\left[A_{0}\times A_{1}\right]}\left\{\gamma_{R-x_{i}}^{\left(T_{1}^{\left(\theta \right)}\right)}\right\}}\left(\gamma \right)$ is given by (A5) or (A6) and $F_{\max_{\left[A_{1}\times A_{2}\right]}\left\{\gamma_{n-x_{i}}^{\left(T_{2}^{\left(\theta \right)}\right)}\right\}}\left(\gamma \right)$ is given by

(A10) $$\begin{array}{cc} \; & F_{\max_{\left[A_{1}\times A_{2}\right]}\left\{\gamma_{n-x_{i}}^{\left(T_{2}^{\left(\theta \right)}\right)}\right\}}\left(\gamma \right)=\sum_{\mu =0}^{A_{1}A_{2}}\frac{{\left(-1\right)}^{\mu }\left(A_{0}A_{1}\right)!}{\mu !\left(A_{0}A_{1}-\mu \right)!}\exp\left(-\frac{\mu \gamma }{\left(\alpha_{i}-\gamma \sum_{j=1}^{i-1}\alpha_{j}\right)\rho_{R}\sigma_{n}^{2}}\right), \end{array}$$

(A11) $$\begin{array}{cc} \; & F_{\max_{\left[A_{1}\times A_{2}\right]}\left\{\gamma_{n-x_{i}}^{\left(T_{2}^{\left(\theta \right)}\right)}\right\}}\left(\gamma \right)=\sum_{\mu =0}^{A_{1}A_{2}}\frac{{\left(-1\right)}^{\mu }\left(A_{1}A_{2}\right)!}{\mu !\left(A_{1}A_{2}-\mu \right)!}\exp\left(-\frac{\mu \gamma }{\alpha_{i}\rho_{R}\sigma_{n}^{2}}\right), \end{array}$$

where Expression (A10) is with i>1 and 0≤γ<αiαi∑j=1i−1αj∑j=1i−1αj and Expression (A11) is with i=1 and γ≥0.

By substituting Expressions (A5), (A6), (A10), and (A11) into Expression (A9), we obtain OP at devices $D_{n}$ in closed-form, as shown in (39).
